# Periocular Vitiligo in Keratoconus: A Case Report and Literature Review

**DOI:** 10.7759/cureus.84690

**Published:** 2025-05-23

**Authors:** Saeed Azizi, Omer Jamall, Zakariya Jarrar, Daniel Gore, Oliver Findl

**Affiliations:** 1 Ophthalmology, Moorfields Eye Hospital, London, GBR; 2 Ophthalmology, Western Eye Hospital, London, GBR; 3 Ophthalmology, Hanusch Hospital, Vienna, AUT

**Keywords:** biomechanics of cornea, cornea and external eye diseases, cornea pathology, keratoconus (kc), ocular complications, periocular diseases

## Abstract

A 21-year-old Asian man developed asymmetrical keratoconus (OD > OS) in the context of right-sided periocular vitiligo. The patient was undergoing topical pimecrolimus treatment for his vitiligo, which caused periocular eye irritation, leading to aggressive, unilateral right-eye rubbing. Subsequently, he developed ocular symptoms such as eye strain-related headaches, aggravated by screen use. There was no relevant past medical history or family history of ocular disease. Corneal topography and keratography confirmed the diagnosis of keratoconus. The patient underwent femtosecond laser-assisted deep anterior lamellar keratoplasty (DALK) in the right eye and corneal cross-linking in the left eye to prevent progression. This case highlights a previously unreported association between periocular vitiligo, pimecrolimus-induced irritation, and subsequent keratoconus. The pathophysiological mechanism is likely multifactorial, involving mechanical stress from chronic eye rubbing and potential alterations in corneal biomechanics due to inflammatory mediators. Given the high prevalence of keratoconus among young patients and the increasing use of topical immunomodulators, this case underscores the need for heightened clinical awareness regarding periocular drug-induced irritation as a modifiable risk factor. It reinforces the importance of patient education on the risks associated with habitual eye rubbing and the need for cautious prescribing of periocular irritants, particularly in individuals at risk for corneal ectatic disorders. To our knowledge, this is the first case report demonstrating that periocular creams with known ocular irritation side effects can exacerbate eye rubbing, which may contribute to the onset and progression of keratoconus. Further research is warranted to explore preventive strategies and alternative therapeutic approaches to mitigate ocular irritation and keratoconus progression.

## Introduction

Cases of keratoconus were alluded to as far back as 1748 by Burchard Mauchart. John Nottingham, a British physician, has been highly accredited for describing distinct features of keratoconus in 1854, differentiating it from other corneal ectasias [1]. Keratoconus is classed as a non-inflammatory ectatic disorder. In keratoconus, the cornea progressively thins and steepens, causing irregular astigmatism and visual impairment. Principal risk factors include eye rubbing, a history of atopy, and a family history [2]. Keratoconus is the most common reason for young patients requiring corneal transplantation [3].

This case report reinforces the importance of patient education on the risks associated with habitual eye rubbing and highlights the necessity for cautious prescribing of topical periocular therapy known to cause eye rubbing, particularly in individuals at risk for corneal ectatic disorders. This is the first case report of keratoconus in a patient with periocular vitiligo who was using topical therapy known to exacerbate eye rubbing, a known risk factor for keratoconus. Written informed consent was obtained from the patient for publication of this case and the included images.

## Case presentation

A 21-year-old male student of Asian origin was first seen in the cornea clinic following an optometry referral due to worsening astigmatism on a background of eye irritation and eye strain. On questioning, the patient reported that the right-sided eye irritation started 8 months ago. He noted this was shortly after commencing topical pimecrolimus therapy prescribed by his dermatologist for a newly formed periocular vitiligo patch located proximal to the right lateral canthus. The use of topical pimecrolimus caused a burning sensation of the overlying skin and led the patient to profusely rub his right eye.

After several months, the patient reported an increasing frequency of eye strain, which was associated with frontal headaches. The headaches would surface after long periods of viewing computer screens and were managed with oral paracetamol as required. The increasing frequency of eye strain prompted an eye check-up at the local optometrist. There was no other past medical history (i.e., atopy), relevant ophthalmic history, or parental consanguinity.

At baseline examination, visual acuity (VA) was noted to be 6/24 OD, improving to 6/9 with pinhole. VA was 6/6 OS. Slit-lamp examination identified Vogt’s striae OU and apical scarring OD. Corneal topography identified bilateral irregular astigmatism and thinning (OD > OS), confirming the diagnosis of keratoconus. Verbal advice was given to avoid rubbing the eyes, and a referral to trial contact lenses was initiated.

**Figure 1:**
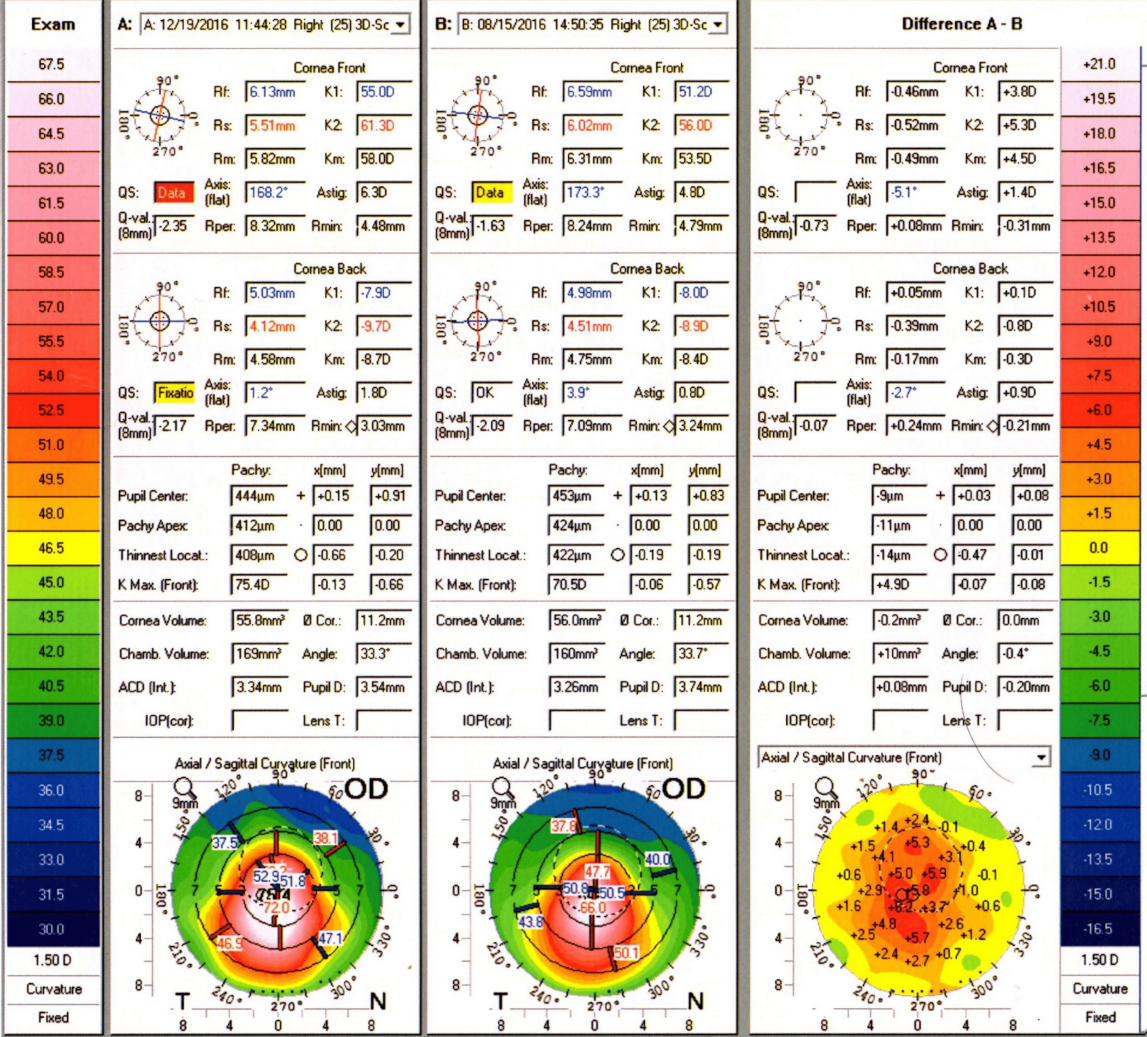
Corneal topography (Pentacam) of the right eye over a four-month period. Corneal topographic (Pentacam) map of the right eye showing significant inferior steepening of the cornea over a four-month period.

**Figure 2:**
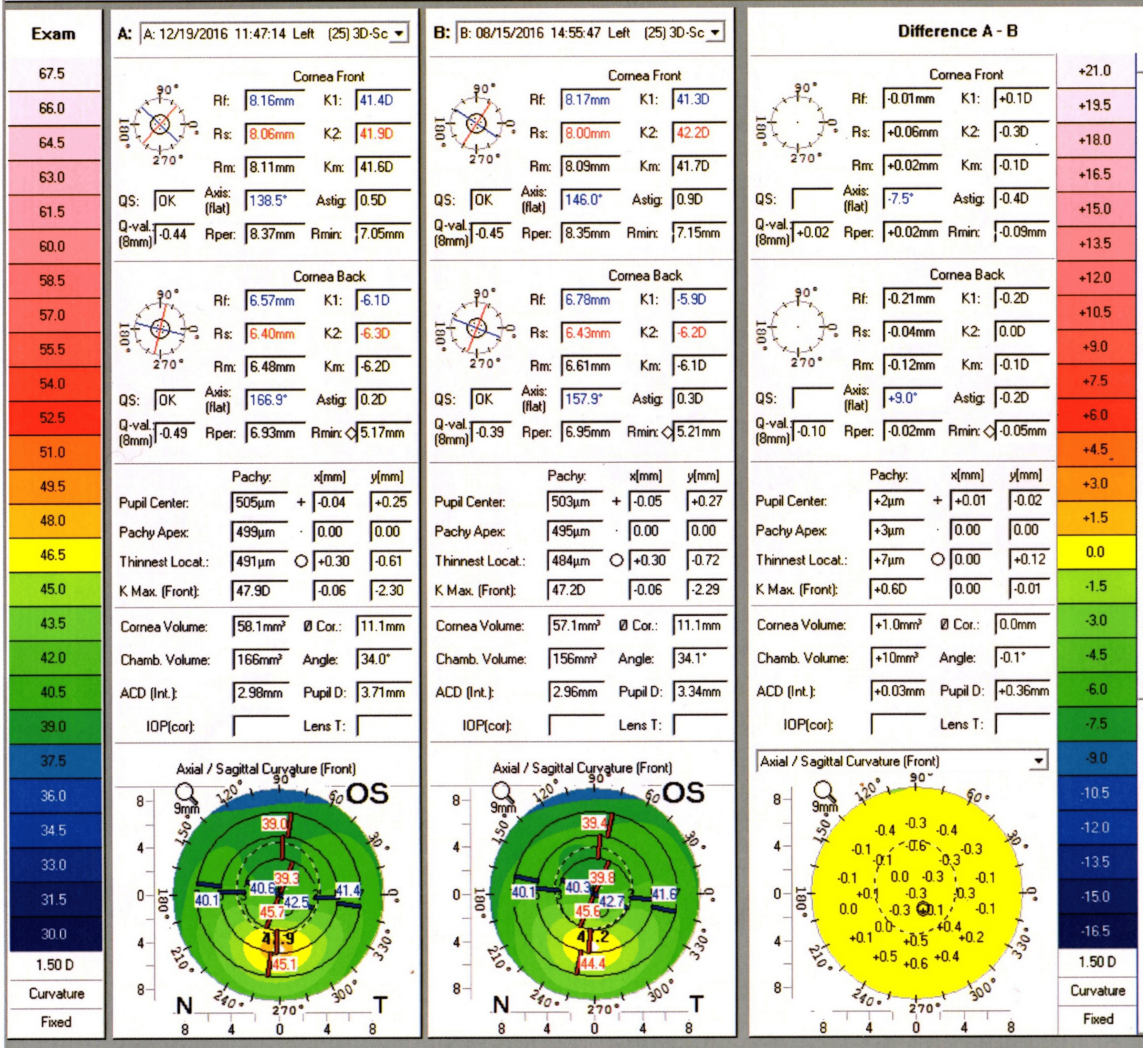
Corneal topography (Pentacam) of the left eye over a four-month period. Corneal topography (Pentacam) results showing the development of early inferior corneal steepening over a four-month period in the left eye.

The patient presented to the cornea clinic four months later. He reported not tolerating the contact lenses but denied further eye rubbing. On examination, VA had deteriorated to 6/36 OD and 6/9 OS. Corneal topography results can be seen for OD (Figure 1) and OS (Figure 2), showing the difference compared to baseline.

The patient initially underwent collagen cross-linking on the left eye to stabilise the corneal shape and prevent further deterioration in vision. For the right eye, a femtosecond laser-assisted deep anterior lamellar keratoplasty (DALK) was performed with interrupted sutures. He was monitored closely and underwent right photorefractive keratectomy (PRK) two years post-DALK, achieving 6/6 vision in both eyes with glasses.

## Discussion

The interplay between external irritants, mechanical stress, and the pathogenesis of keratoconus remains an area of ongoing investigation. An association between keratoconus and vitiligo has yet to be established. This case highlights a potentially underrecognized iatrogenic factor, irritation from periocular medications. In this instance, the use of pimecrolimus, known for causing burning and irritation, likely triggered eye rubbing, a well-established risk factor for keratoconus progression [4]. Calcineurin inhibitors are well-established and effective treatments for periocular dermatitis, and at-risk patients should be carefully counselled regarding the importance of avoiding eye rubbing.

An association between keratoconus and eye rubbing has been postulated as far back as 1940 [5]. The prevalence of eye rubbing in keratoconus patients is higher than in healthy individuals [6-8]. A longer duration of eye rubbing has been observed in patients with keratoconus compared to both the general population and those with infective or allergic ophthalmic conditions [9-10]. A case-control study by Bawazeer AM et al. identified through multivariate analysis that eye rubbing was a significant predictor of keratoconus and concluded that eye rubbing is the most significant cause of keratoconus [11]. A comparable case-control study carried out by Weed KH et al. showed that although 48% of patients with keratoconus reported rubbing their eyes ‘frequently or a great deal’, 39% of the controls who did not have keratoconus also reported rubbing their eyes to the same extent. The same study discovered that some patients had keratoconus without a history of eye rubbing, which highlights the potential role of genetic and/or anatomical predispositions [12].

Similar to our reported case, other case reports have highlighted that the more severely affected eye in keratoconus was the one rubbed more vigorously [13-14]. A case series by Rabinowitz YS et al. identified that out of 8 patients included, 6 had more severe keratoconus on the same side as their dominant hand [15]. A similar correlation was observed among patients who reported aggressive eye rubbing in a study involving 53 patients [10]. In our case, the patient was right-handed and reported rubbing the right eye more aggressively due to the irritation, which may have caused more rapid keratoconus progression. However, the retrospective nature of the eye-rubbing history, absence of genetic or biomechanical testing, and inability to exclude other contributing factors to asymmetry may limit the strength of this association.

Currently, some researchers view eye rubbing as a necessary ‘first-hit’ in the clinical sequelae of keratoconus [16]. Various mechanisms have been proposed for how eye rubbing can cause keratoconus: shear forces altering the corneal shape, temperature changes influencing the corneal structure, raised intraocular pressure causing trauma to keratocytes, and mediators being released by keratocytes as a result of microtrauma [6,17]. This may help explain why keratoconus continues progressing in some patients despite the cessation of eye rubbing.

## Conclusions

This case adds to the growing body of evidence linking eye rubbing with keratoconus progression and underscores the importance of recognizing potential contributors to eye rubbing. While periocular treatments may cause irritation in some patients, careful prescribing and patient education can help minimize unnecessary eye rubbing and its associated risks. Increased awareness among healthcare providers can aid in the early identification of at-risk individuals and support preventative strategies to reduce the likelihood of keratoconus progression.
